# Interpersonal Neural Synchronization Predicting Learning Outcomes From Teaching-Learning Interaction: A Meta-Analysis

**DOI:** 10.3389/fpsyg.2022.835147

**Published:** 2022-02-28

**Authors:** Liaoyuan Zhang, Xiaoxiong Xu, Zhongshan Li, Luyao Chen, Liping Feng

**Affiliations:** ^1^College of Chinese Language and Culture, Beijing Normal University, Beijing, China; ^2^School of Foreign Languages and Literature, Beijing Normal University, Beijing, China; ^3^Max Planck Partner Group, College of Chinese Language and Culture, Beijing Normal University, Beijing, China

**Keywords:** interpersonal neural synchronization, teaching-learning interaction, learning outcomes, meta-analysis, fNIRS, hyperscanning

## Abstract

In school education, teaching-learning interaction is deemed as a core process in the classroom. The fundamental neural basis underlying teaching-learning interaction is proposed to be essential for tuning learning outcomes. However, the neural basis of this process as well as the relationship between the neural dynamics and the learning outcomes are largely unclear. With non-invasive technologies such as fNIRS (functional near-infrared spectroscopy), hyperscanning techniques have been developed since the last decade and been applied to the field of educational neuroscience for simultaneous multi-brain scanning. Hyperscanning studies suggest that the interpersonal neural synchronization (INS) during teaching-learning interaction might be an ideal neural biomarker for predicting learning outcomes. To systematically evaluate such a relationship, this meta-analysis ran on a random-effects model on 16 studies with 23 independent samples (effect sizes). Further moderator analyses were also performed to examine the potential influences of the style, mode, content, and the assessment method of learning outcomes. The random-effects modeling results confirmed a robust positive correlation between INS and learning outcomes. Subsequent analyses revealed that such relationship was mainly affected by both interaction style and mode. Therefore, the present meta-analysis provided a confirmatory neurocognitive foundation for teaching-learning interaction, as well as its relation to the learning outcomes, consolidated future learning and teaching studies in various disciplines including second language education with a firm methodological reference.

## Introduction

In school education, teaching-learning interaction is deemed as a core process in the classroom (Rodriguez, 2013; Rodriguez and Solis, 2013). Teaching-learning interaction means that in classroom settings, the process of information transmission during which feedback is carried out by both teachers and learners (Wagner, 1994) for the purpose of learners’ constructing the correct meaning of the learning content (Wang et al., 2014). Such a teaching-learning interaction process is complicated, including negotiation of meaning, feedback, clarification to facilitate knowledge representation sharing, and mutual understanding (Long, 1996). Underlying this interaction process in various disciplines, similar cognitive processes might be involved and the corresponding neural dynamics might be shared for being congruently indicative of the interaction/learning outcomes (Jiang et al., 2021). At the behavior level, throughout this dynamic interactive process, teachers need to both monitor their own teaching at all times and to evaluate learners’ learning status so as to adjust the teaching content to optimize teaching efficacy (Kline, 2015). Under the cognitive perspective, teaching-learning interaction involves the sub-processes and the mechanisms of knowledge transmission, in which both sides actively exchange information to construct a shared knowledge representation (Strauss et al., 2014). As for the neural correlates underlying teaching-learning interaction, single-brain signals were collected *via* imaging techniques such as fMRI (functional magnetic resonance imaging) for spatial localization (e.g., Ruge and Wolfensteller, 2010). Nevertheless, it is noteworthy that fMRI is not suitable/practical for detecting the neural dynamics during the online interaction process across multiple participants. With the advent of the neuroimaging technologies such as fNIRS (functional near-infrared spectroscopy) and EEG (electroencephalography) featuring strong adaptability and high ecological validity, researchers have developed the hyperscanning approach, a technique for measuring brain activities from two or more participants simultaneously (Montague et al., 2002; Cui et al., 2012; Babiloni and Astolfi, 2014), making it possible to investigate the online teaching-learning interaction. These studies consistently discovered the interpersonal neural synchronization (INS) for successful interaction, which was assumed to be a neural biomarker for predicting the learning outcomes from teaching-learning interaction. Therefore, the present study focused on applying a meta-analysis to the relationship between INS and learning outcomes typically found in published educational neuroscience research, and on explaining any variance in such a relationship attributable to different features of the studies. The results of the present study may shed light on future teaching-learning interaction studies especially in the field of second language education with methodological references.

### Interpersonal Neural Synchronization

Interpersonal neural synchronization shows the brain signal correlation or coherence in time, space and/or frequency dimensions between the communicating sides (see Li, 2018 for details). Through the transmission of language information, INS essentially reflects the shared semantic and predictive representation between individuals. By sharing representations, the individuals can achieve mutual understanding and establish interpersonal relationships (Jiang et al., 2021). Commonly used calculation methods for INS include WTC (Wavelet Transform Coherence, e.g., Zheng et al., 2018; Pan et al., 2020) and Pearson Correlation Coefficient (e.g., Holper et al., 2013; Takeuchi et al., 2017). Besides, researchers also use GCA (Granger Causality Analysis, Granger, 1969) to investigate the causal relationship between the brain signals of teachers and learners (e.g., Pan et al., 2018).

Moreover, INS seems to be housed in various task-related brain areas, including the PFC (prefrontal cortex, e.g., Holper et al., 2013; Liu et al., 2019) for integrating information about oneself and others (Raposo et al., 2011), IFC (inferior frontal cortex, e.g., Pan et al., 2018) associated with both production and comprehension of both language and action (Gallese, 2003), STC (superior temporal cortex, Zheng et al., 2018) for social perception and action observation (Thompson and Parasuraman, 2012), and TPJ (temporal-parietal junction, Pan et al., 2021) for mentalizing and interpersonal prediction (Carter et al., 2012). The abovementioned brain areas have different functions in teaching-learning interaction. With occurrences in various brain areas for predicting different cognitive task performances, the INS *per se* might reflect a particular manner in which neural systems of teachers and learners efficiently communicate with each other to construct a highly shared knowledge representation so as to achieve successful interaction.

### The Relationship Between Interpersonal Neural Synchronization and Learning Outcomes

Previous studies have revealed the correlation between INS and successful verbal communication (e.g., Stephens et al., 2010; Silbert et al., 2014). The hyperscanning studies in educational neuroscience propose that INS might be a critical biomarker for predicting the learning outcomes, i.e., the stronger INS (teacher-learner or learner-learner), the better learners’ learning performances (Pan et al., 2018; Zheng et al., 2018; Liu et al., 2019; Nguyen et al., 2021). For instance, Zheng et al. (2018) used fNIRS to simultaneously collect brain signals of teachers and learners, and identified a significant INS when teachers’ brain activities in the TPJ preceded that of the learners in the STC by 10 s, which was highly correlated with the learning outcomes. By analyzing the teaching behaviors, they proposed that this time-lagged INS might be associated with teachers’ predictions on the answers from the learners. By employing EEG hyperscanning, Davidesco et al. (2019) found that INS among learners could successfully predict the performances of both the immediate and the delayed post-tests. Nguyen et al. (2021) used fMRI to record the brain signals of a teacher recording a teaching video, and then that recorded video was played to the learners. The result showed that the teacher-learner INS was highly correlated with the learners’ performances. Further evidence demonstrated that the increase of the INS induced by tDCS (transcranial direct current stimulation) or tACS (transcranial alternating current stimulation) application over the task-related brain areas such like TPJ and IFC could significantly improve the learning outcomes (Zheng, 2019; Pan et al., 2021). These findings indicated that such brain regions might play a causal role in influencing the quality of teaching and learning through INS.

However, although a relatively large body of studies mentioned above found a positive relationship between INS and learning outcomes (e.g., Dikker et al., 2017; Zheng et al., 2018), Bevilacqua et al. (2019) failed to find reliable correlations between the student-student INS and the post-testing scores (i.e., the learning outcome). Moreover, within the same experiment, even though both communicating sides interact with each other, the relationship between INS and learning outcomes might be affected by several moderators such as interaction style, mode, and content (e.g., Pan et al., 2018; Liu et al., 2019; see Section “Moderators for the Relationship Between Interpersonal Neural Synchronization and Learning Outcomes” for details), and this means that interaction alone could not guarantee a positive relationship between INS and learning outcomes. Therefore, the robustness of the relationship between INS and learning outcomes during teaching-learning interaction remains debatable, and a systematic meta-analysis is necessary for investigating under which condition could such a positive relationship be identified.

### Moderators for the Relationship Between Interpersonal Neural Synchronization and Learning Outcomes

Based on previous studies, the relationship between INS and learning outcomes might be moderated by the following factors. First, the interaction style—whether both sides interact under a face-to-face condition—might affect the INS-learning-outcome relationship. Most studies have revealed that only under the face-to-face teaching condition could INS predict the learning efficiency (Dikker et al., 2017; Zheng et al., 2018; Liu et al., 2019). This condition might facilitate the integration of the multi-modal information including online speech, facial expression, gestures, and eye contacts. But some studies also identified a significant relationship between INS and learning outcomes under the non-face-to-face condition (Meshulam et al., 2021; Nguyen et al., 2021), and thus the interaction style awaited further explorations.

Second, the interaction mode was also reported to have a significant moderating effect, such as: (a) interactive teaching allowing high frequency of turn-taking versus lecturing with comparatively low frequency of turn-taking (Zheng et al., 2018); (b) part learning method emphasizing the interaction (i.e., turn-taking) frequency for each unit during teaching versus whole learning method with constrained turn-taking behaviors permitted only after the whole teaching process (Pan et al., 2018); (c) scaffolding approach containing multiple interactions with high turn-taking frequencies versus explanation-based approach under which teachers introduced and interpreted the knowledge unidirectionally to the learners with very limited turn-taking chances (Pan et al., 2020). In each pair of these examples, the former mode contained “high turn-taking frequency,” whereas the latter one contained “low turn-taking frequency,” and the former mode consistently led to a significant correlation between INS and learning outcomes.

Third, the interaction contents utilized by previous studies could be classified into three categories: the conceptual knowledge (e.g., Nozawa et al., 2019; Pan et al., 2020; Meshulam et al., 2021; Nguyen et al., 2021), the mathematical knowledge (e.g., Zheng et al., 2018; Liu et al., 2019; Pan et al., 2021), and the singing skills for learning a song (Pan et al., 2018, 2021). These contents could roughly correspond to concept learning, rule learning, and skill learning–three distinct learning classes, so interactive content might serve as a potential moderator. One sound explanation is that when the content is relatively abstract, the lecturing mode with low frequency of turn-taking seems to contribute to a better learning performance (Hein et al., 2012, but cf. Aitkin et al., 1981). Currently, we are unaware of direct investigations on the moderating effect of the interaction content on the INS-learning-outcome relationship.

Last, the assessment method of the learning outcomes might also be a potential moderator. In the studies included in the present meta-analysis, besides the tests (e.g., post-testing) of learning performance, questionnaires of learners’ interactive experience and emotional state (e.g., teacher-learner affiliative bond, cognitive load, class participation, teaching satisfaction) were also employed to assess the interaction quality. Both the test scores and interaction quality evaluation scores could serve as assessments of the learning outcomes, but they were not necessarily correlated with INS. For example, Bevilacqua et al. (2019) found that the INS was significantly correlated with the interaction quality evaluation scores rather than the post-testing results. Thus, the assessment method of teaching-learning interaction should be brought to attention when inspecting the relationship between INS and learning outcomes.

To sum up, studies in the last decade have provided sufficient samples (i.e., effect sizes), pointing to the fact that the INS might be a reliable biomarker for predicting the learning outcomes. Such a relationship might be further moderated by several factors. Therefore, the present meta-analysis synthetically investigated the INS-learning-outcome relationship and its possible moderators on the basis of the existing empirical studies.

### Research Aim and Working Hypothesis

We expect that the present study will provide confirmatory evidence for the neural mechanisms of teaching-learning interaction, and identify the key moderators affecting the INS-learning-outcome relationship in a systematic fashion. Based on the existing literature, this study proposes the following hypotheses (H):

H1: There will be a significant correlation between INS and learning outcomes.
H2: Interaction style, mode, content, and assessment method of learning outcomes will be significant moderators influencing the relationship between INS and learning outcomes.

## Methods

The present study was conducted by routinely following Lipsey and Wilson (2001) and Higgins and Green (2011).

### Literature Search and Selection

The literature search focused only on English-language literature in *PubMed* and *Web of Science*. The time range was determined by the publication date of the literature from December 2013 to December 2021. It is noteworthy that we defined the starting time point for the search as when Holper et al. (2013) published the first study applying the hyperscanning techniques into the actual teaching-learning interaction (see also Li (2018) for a similar comment). The search terms were “hyperscanning OR interpersonal neural synchronization OR inter-brain coherence OR inter-brain connectivity OR inter-brain correlation OR inter-brain synchronization OR interpersonal brain synchronization” AND “teach OR learn OR teacher OR learner OR class OR knowledge OR education.” We also returned to the references cited in the previous studies and re-searched the literature in Google Scholar to avoid overlooking other related studies. Consequently, a pool of 212 studies was established for the subsequent screening.

**FIGURE 1:**
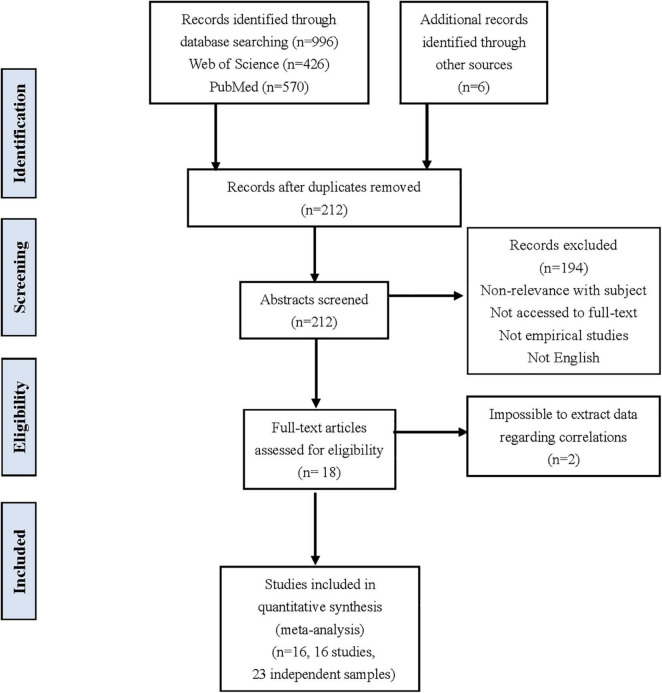
The literature search and selection procedure of the current meta-analysis.

The literature selection criteria were as following: (a) only included empirical studies (i.e., studies with experiments); (b) the studies selected investigated the relationship between the INS of either teacher-to-learner or learner-to-learner, should include the pre- and post-testing scores in the learning outcomes, or the interaction quality evaluation scores reflecting the subjective feelings of the two sides during interaction; (c) these studies reported the sample sizes and with at least one statistic analysis such as the correlation coefficient (i.e., the *r-*value), the *F*-value, the *t*-value, and the regression coefficient (i.e., the β-value), that could reflect the relationship between INS and learning outcomes; (d) the articles were published in English-language journals; (e) only the more comprehensive study was selected from the studies with repeated data. After the screening following these abovementioned criteria, 16 studies with 23 independent samples were kept, in line with the requirement of meta-analysis (Nahman-Averbuch et al., 2018). Detailed analysis information about these studies such as imaging techniques and calculation methods of INS could be found in the Supplementary Material. The literature search and selection procedure were summarized in Figure 1.

### Variable Coding

The current meta-analysis adopted the interaction style, mode, content, and the assessment method of the learning outcomes as four potential factors for the moderating analyses. For each study, we encoded these factors with the authors, publication dates, sample sizes, and the effect sizes. To note, (a) the extraction of effect size was based on each independent sample, and therefore should be encoded only once, and if multiple independent samples were analyzed in the same literature, the effect size was encoded separately for each independent sample; (b) the effect size was also extracted and encoded only once for each moderator. The encoded information was listed in Table 1. Interested readers may also refer to the original literature coding table in Supplementary Material for detailed coding information such as interaction style, interaction mode, interaction content, assessment method, frequency window, target brain area/channel, measurement system (like fNIRS and EEG), and (communication) language.

**TABLE 1:** Descriptions and characteristics of studies investigating the relationship between INS and learning outcomes.

Studies	Sample size	Interaction style	Interaction mode	Interaction content	Assessment of learning outcomes	Effect size (*r*)
Dikker et al., 2017	12	FTF&NFTF	HTF&LTF	Conceptual knowledge	IQ	0.595
Pan et al., 2018	12	FTF	HTF	Song learning	post	0.636
Pan et al., 2018	12	FTF	LTF	Song learning	post	−0.200
Zheng et al., 2018	60	FTF&NFTF	HTF&LTF	Mathematical knowledge	post vs. pre	0.510
Cohen et al., 2018	18	/	/	Conceptual knowledge	post vs. pre	0.494
Liu et al., 2019	17	FTF	LTF	Mathematical knowledge	IQ	0.610
Liu et al., 2019	17	NFTF	LTF	Mathematical knowledge	IQ	−0.080
Liu et al., 2019	17	FTF	LTF	Mathematical knowledge	post	0.730
Liu et al., 2019	17	NFTF	LTF	Mathematical knowledge	post	−0.004
Nozawa et al., 2019	32	FTF	LTF	Conceptual knowledge	IQ	0.330
Bevilacqua et al., 2019	12	FTF&NFTF	LTF	Conceptual knowledge	IQ	0.382
Bevilacqua et al., 2019	12	FTF&NFTF	LTF	Conceptual knowledge	post	0.130
Davidesco et al., 2019	31	FTF	LTF	Conceptual knowledge	post	0.520
Zhu et al., 2019	16	/	/	/	IQ	0.622
Pan et al., 2020	24	FTF	HTF	Conceptual knowledge	post vs. pre	0.655
Pan et al., 2020	24	FTF	LTF	Conceptual knowledge	post vs. pre	−0.210
Nguyen et al., 2021	20	/	/	Conceptual knowledge	post vs. pre	0.673
Sun et al., 2020	34	/	/	/	/	0.650
Sun et al., 2020	34	/	/	/	/	0.580
Zheng et al., 2020	60	FTF&NFTF	HTF&LTF	Mathematical knowledge	IQ	0.310
Pan et al., 2021	16	FTF	HTF	Mathematical knowledge	post vs. pre	0.510
Meshulam et al., 2021	24	/	/	Conceptual knowledge	post vs. pre	0.270
Zhu et al., 2021	24	FTF	HTF	Conceptual knowledge	post vs. pre	0.570

*FTF, face-to-face; NFTF, non-face-to-face; HTF, High turn-taking frequency; LTF, low turn-taking frequency; Post, post-testing scores; Post vs. Pre, comparison between post-testing and pre-testing scores; IQ, interaction quality evaluation scores.*

*“/” indicates the coding is not applicable for the sample.*

*Interaction style and mode for offline scanning studies were not coded.*

*In addition, the study of Sun et al. (2020) was only conducted in the overall effect size analysis because the experiment setting is different from others and the moderators are not applicable, but the effect size (r) reflects the relationship between INS and teacher-learner interaction outcome.*

### Data Synthesis and Analyses

In accordance with Borenstein et al. (2009), the present meta-analysis adopted the INS-learning-outcome correlation coefficient (*r*) as the index for effect size, and employed CMA3.3 (Comprehensive Meta-Analysis Version 3.3) (Borenstein et al., 2009) for both the overall effect size and the moderating effect sizes.

To specify, “effect size” is a measure of experimental effect strength or variable correlation strength (Snyder and Lawson, 1993), which is barely affected by the sample size. According to the statistics, the effect sizes could be classified into three families (Borenstein et al., 2009): difference family (e.g., Cohen’s *d*, Hedge’s *g*), correlation family (e.g., Pearson’s *r*, *R*^2^), and category family (e.g., odds ratio, risk ratio). Therefore, the correlation coefficient (i.e., Pearson’s *r*) is a kind of effect size.

A meta-analysis could accumulate the effect sizes reported by the previous studies so as to compare the research results in a systematic fashion (Hunter and Schmidt, 2004). In the present study, all the studies focused on the relationship between INS and learning outcomes, and reported their corresponding correlation coefficients. Therefore, the correlation coefficient is deemed as the most reliable effect size for statistical analysis when the studies focused on the variable correlations (Borenstein et al., 2009). Similarly, a number of studies also used correlation coefficient as effect size in their meta-analyses (Le et al., 2018; Lei et al., 2020; Vahedi and Zannella, 2021). Thus, the *F*-value, the *t*-value and the β-value reported in some studies were also converted to the *r*-values [$r=\sqrt{\frac{F}{F+d\,f\,e}}$; *r* = $\sqrt{\frac{t^{2}}{t^{2}+d\,f}}$; *r = β* × 0.98 + 0.05 (β ≥*0*); *r = β* × 0.98*-* 0.05 (β <*0*) (β∈(−0.5, 0.5))] (Peterson and Brown, 2005). In case of the result deviation, several effect sizes in the same study would be synthesized *via* the “combination algorithms” implemented in CMA3.3. More specifically, if a single study reported several effect sizes generated from its multiple experiments or experimental conditions, this might increase the weight of this study in meta-analysis and lead to a result bias (Borenstein et al., 2009). By following Rivis and Sheeran (2003), Lei et al. (2020), and Asegid and Assefa (2021), in the present study we *combined* the effect sizes under certain conditions of no interest as a single unit within the same study. For instance, since the present study focused on the INS and its relationship with the learning outcomes regardless of the specific brain activation patterns (i.e., brain regions), correlation coefficients between INS and learning outcomes in different brain regions or channels under a certain condition within the same study were of no interest, and thus they were combined as a *synthesized* effect size.

The combination of the effect sizes was realized by computing the averaged weighted correlation coefficient of independent samples (Rivis and Sheeran, 2003; Lei et al., 2020). We used the CMA3.3 software to combine the effect sizes in the following steps: (a) Fisher-*z* transformation from the correlation coefficients to *Z*-values. (b) Combining the *Z*-values *via* the inverse variance weights, a typical method used in meta-analyses for combining the effect sizes by aggregating two or more random variables, each of which was weighted in inverse proportion to its variance, so as to minimize the variance of the sum (Lipsey and Wilson, 2001). (c) The calculation results were converted into “*r*” again with the inverse *Zr* transformation. To note, the original literature coding table was provided as the Supplementary Material for specifying that the effect sizes under the same serial number in this table were combined.

Given that the studies included in the present research may be affected by several moderators, it was more appropriate to choose the random-effects model (Borenstein et al., 2009). We selected *Q*-statistic and *I*^2^ statistics for further verification according to Huedo-Medina et al. (2006). The *Q*-statistic was calculated to verify the heterogeneity among effect sizes, *p* < 0.05. The *I*^2^ was also computed to quantify heterogeneity, which represents the percentage of variation across studies. *I*^2^ values of 25, 50, and 75% indicate low, moderate, and high levels of heterogeneity, respectively. If an effect size is significantly heterogeneous and not at a low level (≥25%), it indicates the reliability of adopting the random-effects model (Higgins and Green, 2011). Regarding the moderator analyses, we grouped the studies into categories and compared the differences among these categories using the *Q*-statistic (Borenstein et al., 2009).

Besides, the experimental overall effect size might be overestimated due to the fact that the significant results were more prone to be published (Rothstein et al., 2005). Thus, the publication bias tests were further performed to evaluate the representativeness in the present meta-analysis, including Fail-Safe (*Nfs*, Rothstein et al., 2005), Egger regression (Egger et al., 1997), and the Trim-and-Fill method (Duval and Tweedie, 2000). In brief, *N*_*fs*_ reflects the number of unpublished studies needed to nullify the effect size of a meta-analysis to a non-significant level (Rothstein et al., 2005). If the *N*_*fs*_ exceeds 5*k* + 10 (*k* = the number of independent effect size in the meta-analysis), we may conclude that the effect size is robust (Rothstein et al., 2005). A significant Egger’s regression test result (*p* < 0.05) is considered to indicate the existence of the publication bias (Egger et al., 1997). The trim-and-fill method provides an estimate of how many studies are missing from the analysis and calculates an adjusted effect size including the filled studies, generating an intervention effect adjusted for publication bias. If the adjusted effect size is different from the original, the overall effect size of the meta-analysis will be considered to have a publication bias (Rothstein et al., 2005).

## Results

### Overall Effect Size and the Homogeneity Test

**FIGURE 2:**
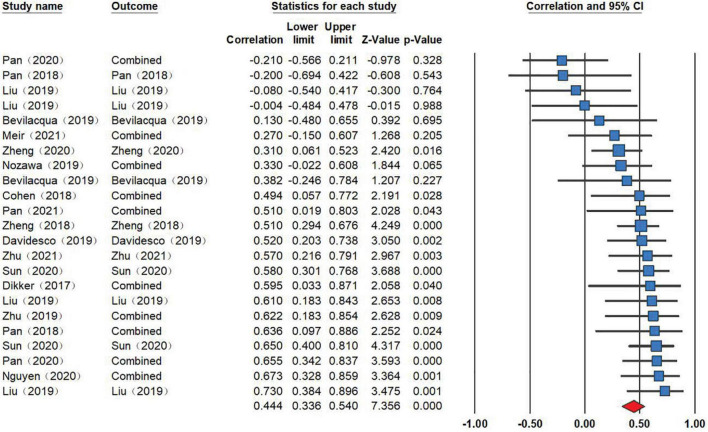
Studies included in the meta-analysis investigating the relationship between INS and learning outcomes (*r* = 0.444, *p* < 0.001, 95% CI [0.34, 0.54]). Effect sizes to the right of the zero mark indicate a positive relationship between INS and learning outcomes, whereas effect sizes to the left of the zero mark indicate a negative relationship between INS and learning outcomes. The middle point of the red filled diamond represents the overall effect size, and the two ends of the long diagonal line of the diamond represent 95% Confident Intervals (CI). The effect size of each independent sample is represented by the blue filled square, and the two ends of the line passing through the square represent 95% CI. The size of the square represents weight (i.e., contribution to meta-analysis). “Combined,” means when one study reported several effect sizes, these effect sizes would be synthesized *via* the “combination algorithms” function in CMA 3.3. To note, recurring studies represent different independent samples.

The random-effects modeling results (see Figure 2) on the 23 independent samples revealed a significant positive correlation between INS and learning outcomes (*r* = 0.444, *p* < 0.001, 95% CI [0.34, 0.54]). *Post-hoc* sensitivity tests were performed by randomly excluding one sample each time, and found that the overall effect size was ranging from 0.429 to 0.472 (>0.300), thus exempted from the influence of the extreme data (Gignac and Szodorai, 2016).

The heterogeneity test for 23 independent samples showed substantial heterogeneity among the samples (*Q* = 40.242; *p* < 0.05; *I*^2^ = 40.361) and suggested that the use of a random-effects model was appropriate. Moderator analyses were then conducted to account for the variance across these studies.

Moreover, according to aforementioned methods of estimating publication bias, we performed a series of tests and further confirmed that the overall effect size of the present meta-analysis was unlikely to be enhanced by the publication bias (Table 2).

**TABLE 2:** Publication bias estimation.

Outcome variable	*N*_*fs*_	Egger’s regression	SE	95%CI	*p*	Effect size (after Trim and Fill)
The relationship between INS and learning outcomes	597	−0.35	0.917	[−2.49, 1.79]	>0.05	0.436

*95% CI indicates 95% confidence intervals of Egger’s intercept.*

### Moderator Analyses Results

The moderator analyses results (Table 3) revealed that both the interaction style and mode would significantly moderate the INS-learning-outcome relationship. The interaction style was a significant moderator for the relationship between INS and learning outcomes (*Q* = 5.764, *p* < 0.05), and the effect size of face-to-face (*r* = 0.455) showed the correlation significance. The interaction mode was a significant moderator for the INS-learning-outcome relationship (*Q* = 5.591, *p* < 0.05), and the effect size of high turn-taking frequency mode (*r* = 0.598) was significant. None of the other two moderators showed a significant effect on the relationship between INS and learning outcomes (interaction content, *Q* = 0.150, *p* > 0.05; assessment method of learning outcomes, *Q* = 0.483, *p* > 0.05).

**TABLE 3:** Moderation results of the categorical moderators (Random effects model).

Categorical moderators	*K*	*r*	*p*	95%CI	Heterogeneity test		
					*Q*_*B*_	*df*	*p*
Interaction style					5.674	1	0.017
Face-to-face	10	0.455	<0.001	[0.25, 0.62]			
Non-face-to-face	2	−0.042	0.824	[−0.39, 0.32]			
Interaction mode					5.591	1	0.018
High turn-taking frequency	4	0.598	<0.001	[0.42, 0.73]			
Low turn-taking frequency	10	0.265	0.031	[0.03, 0.48]			
Interaction content					0.150	2	0.928
Song learning	2	0.267	0.565	[−0.58, 0.84]			
Conceptual knowledge	11	0.425	<0.001	[0.26, 0.57]			
Mathematical knowledge	7	0.406	<0.001	[0.20, 0.58]			
Assessment method of learning outcomes					0.483	2	0.785
Post-testing scores	6	0.368	0.029	[0.04, 0.63]			
Post vs. pre-testing scores	8	0.458	<0.001	[0.26, 0.62]			
Interactive quality scores	7	0.379	<0.001	[0.21, 0.53]			

*Heterogeneity Q, indicates the level of heterogeneity across studies; K, number of studies; CI, Confidence Intervals.*

*Given some p-values are too small, we used “<0.001” to report the significance.*

## Discussion

To our best knowledge, this is the first meta-analysis aiming to examine systematically on the relationship between INS and learning outcomes. The current results converged on that INS might be an ideal neural biomarker for predicting the learning outcomes. Furthermore, INS-learning-outcome relationship might be significantly influenced by interaction style and mode, rather than the content and the assessment method.

### Interpersonal Neural Synchronization: A Biomarker for the Learning Outcomes Prediction

Supported by plenty of previous studies (Holper et al., 2013; Pan et al., 2018, 2020; Zheng et al., 2018; Meshulam et al., 2021; Nguyen et al., 2021), this current meta-analysis confirmed a positive relationship between INS and learning outcomes, further demonstrating that INS, as a neural biomarker in the context of social interaction during school education, might play a critical role in predicting learning outcomes, thus corresponding to Dikker et al. (2017).

A key question is why a stronger INS is potentially valid for predicting a better learning outcome. According to Nozawa et al. (2019) and Jiang et al. (2021), in the process of verbal communication, individuals always seek to minimize the differences between themselves and their peers in the aspects of gestures, semantics, syntax, and mental states, which will lead to the improvement of similarity and harmony between individuals, thus enhancing the synchronization of the neural activities. Accordingly, in the context of school education, we reasoned that in order to achieve a successful learning result, teachers and learners, or learners and their classmates might endeavor to reduce the differences on sub-processes like negotiation of meaning, feedback, clarification and so on, forming together a shared knowledge representation reflected by the increase of INS. Studies using non-invasive stimulation techniques further demonstrated a causal role of INS in better collaboration between the interaction parties (Zheng, 2019; Pan et al., 2021). Moreover, it has been proposed that the INS might predict the learning/interaction outcomes at different levels (Jiang et al., 2021). Basic acoustic features might trigger the INS during interactive speech processing. INS at a higher-level interaction might reflect mutual understanding, and at the ultimate level of social interaction, might be indicative of relationship establishment and maintenance (see Jiang et al. (2021) for a detailed description of this hierarchical model for verbal communication). Nevertheless, despite that INS is able to occur at different levels, this neural biomarker might be critical to reveal the degree of the shared knowledge representation at a certain level, thus being informative to predict the interaction results of this level in particular.

### Critical Factors in Moderating the Interpersonal Neural Synchronization-Learning-Outcome Relationship

The current meta-analysis revealed that the interaction style and mode might be critical for moderating the relationship between INS and learning outcomes. Compared with the non-face-to-face (such as back-to-back) style, interaction in a face-to-face manner could provide teachers and learners with more visual inputs, so INS was mainly contributed by audiovisual information integration (Jiang et al., 2012). Non-linguistic visual information such as facial expressions, gestures, and eye-contacts might be crucial for the establishment and maintenance of the emotion and feelings for both sides, and teachers might extract information from learners’ facial expressions to adjust the pedagogical processes (Liu et al., 2019). Therefore, interaction style is important for the dynamic integration of various information from both auditory and visual modalities, as well as for forming more harmonious interpersonal relationships.

As for the interaction mode, high turn-taking frequency mode is reasonably more influential on enhancing INS. To illustrate, Pan et al. (2018) observed that both behavioral performance and INS increased particularly when the learning experience entailed high frequency of turn-taking to support a more active interaction (i.e., the part learning method). This finding is consistent with previous evidence regarding the verbal communication. Jiang et al. (2012) found a significant increase of INS during a fact-to-face dialog between partners but not during a face-to-face monolog; moreover, the INS between partners during the face-to-face dialog resulted primarily from the direct interactions underlying the successful communication, featured by the high turn-taking frequency. The turn-taking behaviors between partners were assumed to play a pivotal role in social interactions through modifying their own actions in response to the continuously changing actions (Dumas et al., 2011). Therefore, INS might be driven by the turn-taking frequencies in teaching-learning interaction. High turn-taking frequency might make the interaction process more active so as to provide both teachers and learners with more opportunities to adjust their behaviors to achieve mutual understanding, thus improving the learning efficiency.

The present study did not identify a significant effect of interaction content. A plausible account is that the teaching materials utilized in these studies, such as the teaching of specific concepts in certain disciplines (e.g., Nozawa et al., 2019; Pan et al., 2020) or simple skills (Pan et al., 2018), might be equally easy to learn. Thus, there is no significant difference in the amount of cognitive resources (in the processes of understanding, memorizing, and prediction) required by teachers and learners, resulting in a non-significant influence on the relationship between INS and learning outcomes. Future studies might utilize the interaction content of different types and complexities for a further investigation.

Furthermore, different assessment methods showed a similar impact on the INS-learning-outcome relationship. Specifically, the assessment methods can be classified into testing scores and interaction quality scores. In particular, testing scores include the scores of pre-tests, post-tests, and delay-tests, which reflect the students’ mastery of the teaching contents (i.e., learning achievements). Interaction quality scores denote the scores of various interaction scales including the evaluations of teacher-student relationship (Zheng et al., 2020), the degree of how much the students favor the class (Nozawa et al., 2019), and of the attention degree (Dikker et al., 2017). These scale scores reflect the subjective feelings and emotional experiences of learners in the process of interaction. In the present study, each assessment method type revealed a significant positive relationship between the INS and learning outcomes. In other words, the significant correlations between INS and learning outcomes are independent of the assessment method types, rendering the assessment method as a non-significant factor in the moderator analysis. In line with Watanabe et al. (2013), we reasoned that since the teaching-learning interaction is a bi-directional complex dynamic process, it is insufficient to merely take the learning performance (i.e., the interaction results) into account. Moreover, the subjective feeling like the emotional experience is also assumed to be crucial for human cognition such as attention accommodation, decision making, problem solving, behavioral executive control, and creativity (Steele and Aronson, 1995), and it might inevitably affect the learning processes, as suggested by the Krashen’s Affective Filter Hypothesis in the field of second language learning (Krashen, 1982). Jiang et al. (2021) also hypothesized that the maintenance of a positive interpersonal relationship could also be crucial for improving the INS and augmenting better learning outcomes. Therefore, our current results further justified that both learning performances, as reflected by the testing scores and subjective feelings/emotional experiences as measured by the interaction quality scores, were all critical for teaching-learning interaction and could serve as sensitive measurements tapping into different aspects during the teaching-learning interaction process, respectively.

It is also noteworthy that for certain moderators, the sample sizes of the studies were relatively small. Due to the fact that using the hyperscanning techniques in the field of educational neuroscience for investigating the teaching-learning interaction is still at an early stage, the emerging studies focusing on aspects such as non-face-to-face style and learning songs are still in the minority. Nevertheless, comparing these studies with others in the moderator analyses could still reveal some statistical trends, though readers should be cautiously bear in mind that it is premature to draw a firm conclusion from the current results of certain moderator analyses, which are limited to the sample sizes.

## Outlook

The present meta-analysis systematically examined the relationship between INS and learning outcomes during the teaching-learning interaction. A significant overall effect size was found for the INS-learning-outcome relationship, confirming that the INS might be a reliable neural biomarker for predicting the learning outcomes. According to the current meta-analysis, INS might reflect the shared knowledge representation between teachers and learners, thus enhancing the mutual understanding and the establishment of interpersonal relationship so as to successfully improve the learning performances. Moreover, subsequent analyses on the potential moderators further demonstrated that both the interaction style and mode might be critical for tuning this relationship.

As with other disciplines of education, interactive and cooperative learning are also crucial for L2 teaching and learning, therefore, the results of this meta-analysis can be applied to investigate the process of L2 teaching-learning interaction. By referring to the results of this meta-analysis, future studies may scrutinize such a relationship and test these L2-specific moderators in a more comprehensive manner by using INS as a biomarker. In the studies recruited, the individuals in the teaching-learning interaction came from the same language background. However, for L2 teaching and learning, the language context is much more complicated, as Pérez et al. (2019) found that the linguistic context type could impact on the INS between speakers and listeners, the distinctions from language typologies and cultures might also affect the relationship between INS and the L2 learning outcomes. Therefore, it is an intriguing topic of great significance in the field of second language education in the future to explore the role of different factors in interpreting and predicting L2 learning outcomes by taking INS as a critical neural marker. And this may illuminate the way of improving L2 learning efficiency by constructing the teaching and learning methods under different circumstances in a more effective fashion.

## Data Availability Statement

The original contributions presented in the study are included in the article/Supplementary Material, further inquiries can be directed to the corresponding author/s.

## Author Contributions

LZ, XX, and LC conducted the meta-analysis. LZ and LC completed the first draft of this manuscript, which was further revised by XX, ZL, and LF. All authors participated in the discussion of the results and the final revision of the manuscript, and revised the manuscript according to the reviewers’ comments.

## Conflict of Interest

The authors declare that the research was conducted in the absence of any commercial or financial relationships that could be construed as a potential conflict of interest.

## Publisher’s Note

All claims expressed in this article are solely those of the authors and do not necessarily represent those of their affiliated organizations, or those of the publisher, the editors and the reviewers. Any product that may be evaluated in this article, or claim that may be made by its manufacturer, is not guaranteed or endorsed by the publisher.
