# Supplementary material for: Interpersonal Neural Synchronization Predicting Learning Outcomes From Teaching-Learning Interaction: A Meta-Analysis
Source: Front Psychol. 2022 Feb 28;13:835147. doi: 10.3389/fpsyg.2022.835147 (PMC8918582; doi:10.3389/fpsyg.2022.835147)
Supplement: Supplementary file 1 [file Table_1.DOCX]

**Supporting Information**

Table S1. Original coding table for the studies included in the current meta-analysis.

|  | **Studies** | **N** | **IS** | **IM** | **Interaction Content** | | **Assessment of Learning Outcomes** | | **Correlation Coefficient** | | **MT** | **CM of INS** | **Frequency Band** | **Brain Areas of INS** | **Teaching Language** |
| --- | --- | --- | --- | --- | --- | --- | --- | --- | --- | --- | --- | --- | --- | --- | --- |
|  |  |  |  |  | **Category** | **Detailed information** | **Category** | **Detailed information** | ***r*** | **Detailed information** |  |  |  |  |  |
| 1 | Dikker  2017 | 12 | FTF  &  NFTF | HTF | conceptual knowledge | conceptual knowledge in neuroscience crash-course | IQ | students’ ratings of the class | 0.61 | the relationship between INS and students' ratings | EEG | TI | / | / | English |
|  | Dikker  2017 | 12 |  |  |  |  |  | group affinity ratings | F=5.95→*r*=0.61 | the relationship between INS and group affinity scores |  |  |  |  |  |
|  | Dikker  2017 | 12 |  |  |  |  |  | group empathy ratings | F=5.71→*r*=0.6 | the relationship between INS and group empathy scores |  |  |  |  |  |
|  | Dikker  2017 | 12 |  |  |  |  |  | student focus ratings | F=4.64→*r*=0.56 | the relationship between INS and student focus scores |  |  |  |  |  |
| 2 | Pan  2018 | 12 | FTF | HTF | song learning | 2 Chinese songs: "The Moon Reflection" (Lyrics: B. Peng, Music: Z. Liu and S. Yan) and "A Tune of Homesickness" (Lyrics: C. Qu, Music: Q. Zheng). These songs entailed simple lyrics and melodies and they were likely to be unfamiliar to the participants. | post | 6 aspects of music performance were evaluated through 7-point scales (i.e., melody, rhythm, lyric, pitch, emotion, and tune). The sum of the judgements made on all 6 aspects (for a given learner) was considered as the index of overall learning performance. | 0.58 | the relationship between average INS and overall learning performance | fNIRS | WTC | 0.07-0.15Hz | bilateral inferior frontal cortex | Chinese |
|  | Pan  2018 | 12 |  |  |  |  |  |  | 0.63 | the relationship between VI-INS (verbal-interaction INS) and overall learning |  |  |  |  |  |
|  | Pan  2018 | 12 |  |  |  |  |  |  | 0.69 | the relationship between VI-INS and pitch performance during observation |  |  |  |  |  |
| 3 | Pan  2018 | 12 | FTF | LTF | song learning | 2 Chinese songs: "The Moon Reflection" (Lyrics: B. Peng, Music: Z. Liu and S. Yan) and "A Tune of Homesickness" (Lyrics: C. Qu, Music: Q. Zheng). These songs entailed simple lyrics and melodies and they were likely to be unfamiliar to the participants. | post | 6 aspects of music performance were evaluated through 7-point scales (i.e., melody, rhythm, lyric, pitch, emotion, and tune). The sum of the judgements made on all 6 aspects (for a given learner) was considered as the index of overall learning performance. | -0.20 | the relationship between average INS and overall learning performance | fNIRS | WTC | 0.07-0.15Hz | bilateral inferior frontal cortex | Chinese |
| 4 | Zheng  2018 | 60 | FTF  &  NFTF | HTF  &  LTF | mathematical knowledge | Numerical reasoning tasks. For instance, the number sequence of “2, 4, _,8, 10, 12” follows the rule that all numbers are even numbers that differ by the constant of 2, so “6” is the correct answer. | post VS. pre | Pre & post-testing: 15 4-choice items were selected from CCSAPAT’s test bank. The outcome was indexed by the change score (pre-test scores were subtracted from post-test scores). | F=19.297→*r*=0.51 | teaching outcome was a significant positive variate of INS between teacher and student | fNIRS | WTC | 0.5-0.7Hz | anterior superior temporal cortex, students' temporal-parietal junction | Chinese |
| 5 | Cohen  2018 | 18 | / | / | conceptual knowledge | Educational videos on topics related to physics and biology | post VS. pre | Four alternative forced-choice questionnaire (9-12 questions) both before and after learning. The outcome was indexed by the change score (pre-test scores were subtracted from post-test scores). | 0.57 | the relationship between INS and test performance in the cohort of intentional | EEG | ISC | / | / | English |
|  | Cohen  2018 | 18 |  |  |  |  |  |  | 0.41 | the relationship between INS and test performance in the cohort of incidental |  |  |  |  |  |
| 6 | Liu  2019 | 21 | FTF | LTF | mathematical knowledge | To calculate conditional probability correctly, teachers would teach students how to use the formula and its variants. | IQ | Perceived teacher-student interaction was assessed on a 5-point Likert-type scale. | 0.61 | the INS was significantly correlated with perceived teacher-student interaction scores | fNIRS | WTC | 0.15-0.31Hz | prefrontal area | Chinese |
| 7 | Liu  2019 | 21 | NFTF | LTF | mathematical knowledge | To calculate conditional probability correctly, teachers would teach students how to use the formula and its variants. | IQ | perceived teacher-student interaction was assessed on a 5-point Likert-type scale. | -0.08 | the INS was not significantly correlated with perceived teacher-student interaction | fNIRS | WTC | 0.15-0.31Hz | prefrontal area | Chinese |
| 8 | Liu  2019 | 21 | FTF | LTF | mathematical knowledge | To calculate conditional probability correctly, teachers would teach students how to use the formula and its variants. | post | The post-testing contains five questions related to the learning content. | 0.73 | the INS was significantly correlated with students' scores | fNIRS | WTC | 0.15-0.31Hz | prefrontal area | Chinese |
| 9 | Liu  2019 | 21 | NFTF | LTF | mathematical knowledge | To calculate conditional probability correctly, teachers would teach students how to use the formula and its variants. | post | The post-testing contains five questions related to the learning content. | 0.004 | the INS was not significantly correlated with students' scores | fNIRS | WTC | 0.15-0.31Hz | prefrontal area | Chinese |
| 10 | Nozawa  2019 | 32 | FTF | LTF | conceptual knowledge | The teacher repeatedly delivered the word, meaning, and example sentence to the student, while trying to memorize everything for themselves. | IQ | mutual attentiveness scores | 0.34 | mutual attentiveness showed significant positive correlation with INS | fNIRS | WTC | / | prefrontal cortex | Japanese |
|  | Nozawa  2019 | 32 |  |  |  |  |  | mutual positivity scores | 0.21 | mutual positivity did not show a significant positive correlation with INS |  |  |  |  |  |
|  | Nozawa  2019 | 32 |  |  |  |  |  | mutual coordination scores | 0.43 | mutual coordination showed significant positive correlation with INS |  |  |  |  |  |
| 11 | Bevilacqua  2019 | 12 | FTF  &  NFTF | LTF | conceptual knowledge | conceptual knowledge in neuroscience crash-course | IQ | student-teacher closeness scores | 0.382 | the correlation between student-teacher closeness and student-teacher INS | EEG | TI | / | / | English |
| 12 | Bevilacqua  2019 | 12 | FTF  &  NFTF | LTF | conceptual knowledge | conceptual knowledge in neuroscience crash-course | post | Students completed a 20-question multiple-choice knowledge quiz immediately after each recording session (six in total) to test retention of core concepts. | F=0.05→*r*=0.04 | the correlation between quiz scores and student-teacher INS | EEG | TI | / | / | English |
| 13 | Davidesco  2019 | 31 | FTF | LTF | conceptual knowledge | conceptual knowledge in science class | post | A short quiz that students answered three topic-related questions. | 0.52 | the relationship between INS and delayed retention scores | EEG | TI | / | / | English |
| 14 | Zhu  2019 | 16 | / | / | / | / | IQ | learning desire scores | 0.74 | INS was significantly correlated with learning desire | EEG | ISC | / | / | Chinese |
|  | Zhu  2019 | 16 |  |  |  |  |  |  | 0.66 | subcomponents INSs was independently correlated with learning desire |  |  |  |  |  |
|  | Zhu  2019 | 16 |  |  |  |  |  |  | 0.58 |  |  |  |  |  |  |
|  | Zhu  2019 | 16 |  |  |  |  |  |  | 0.47 |  |  |  |  |  |  |
| 15 | Pan  2020 | 24 | FTF | HTF | conceptual knowledge | The sets centered around the concepts of reinforcement and transfer. These concepts were chosen from a classic national standard textbook (Educational Psychology: A Book for Teachers). These two concepts belong to the similar topic (i.e., learning psychology) and occupy a similar instructional period (i.e., 1~2 sessions). | IQ | Three question types (i.e., definitions, true-false items, simple answer questions) were evaluated. The sum of the judgments made on all three question types (for a given learner) was considered as the index of overall learning performance. Learning outcomes were quantified as the difference pre-testing scores and post-testing scores. | 0.65 | INS was positively correlated with learning outcomes | fNIRS | WTC | 0.45–0.57 Hz and 0.17–0.27 Hz | prefrontal regions | Chinese |
| 17 | Pan  2020 | 24 | FTF | LTF | conceptual knowledge | The sets centered around the concepts of reinforcement and transfer. These concepts were chosen from a classic national standard textbook (Educational Psychology: A Book for Teachers). These two concepts belong to the similar topic (i.e., learning psychology) and occupy a similar instructional period (i.e., 1~2 sessions) | post VS. pre | Three question types (i.e., definitions, true-false items, simple answer questions) were evaluated. The sum of the judgments made on all three question types (for a given learner) was considered as the index of overall learning performance. Learning outcomes were quantified as the difference pre-testing scores and post-testing scores. | 0.24 | INS was not positively correlated with learning outcomes | fNIRS | WTC | 0.45–0.57 Hz and 0.17–0.27 Hz | prefrontal regions | Chinese |
|  | Pan  2020 | 24 |  |  |  |  |  |  | -0.18 | INS was not positively correlated with learning outcomes |  |  | 0.45–0.57 Hz and 0.17–0.27 Hz | superior temporal cortex | Chinese |
| 18 | Nguyen  2020 | 20 | / | / | conceptual knowledge | conceptual knowledge related to fMRI | post VS. pre | pre-testing: multiple choice 25-question quiz; post-testing: the same 25-question quiz. Normalized score = (Posttest - Pretest) * mean (Posttest + Pretest) | 0.663 | seven regions with a significant correlation between teacher-student INS during the lesson and learning outcomes | fMRI | ISC | / | posterior cingulate cortex | English |
|  | Nguyen  2020 | 20 |  |  |  |  |  |  | 0.626 |  |  |  |  | right precuneus |  |
|  | Nguyen  2020 | 20 |  |  |  |  |  |  | 0.751 |  |  |  |  | left precuneus |  |
|  | Nguyen  2020 | 20 |  |  |  |  |  |  | 0.634 |  |  |  |  | superior occipital gyrus |  |
|  | Nguyen  2020 | 20 |  |  |  |  |  |  | 0.755 |  |  |  |  | dorsal precuneus |  |
|  | Nguyen  2020 | 20 |  |  |  |  |  |  | 0.633 |  |  |  |  | left lateral occipital complex |  |
|  | Nguyen  2020 | 20 |  |  |  |  |  |  | 0.619 |  |  |  |  | right hV4 |  |
| 19 | Sun  2020 | 34 | / | / | / | / | / | / | 0.65 | the relationship between INS and average accuracy rate | fNIRS | WTC | 0.04-0.16Hz | the left dorsolateral PFC | / |
| 20 | Sun  2020 | 34 | / | / | / | / | / | / | 0.58 | the relationship between INS and perspective-taking ability | fNIRS | WTC | 0.04-0.16Hz | the left dorsolateral PFC | / |
| 21 | Zheng  2020 | 60 | FTF  &  NFTF | HTF  &  LTF | mathematical knowledge | Numerical reasoning tasks. For instance, the number sequence of “2, 4, _,8, 10, 12” follows the rule that all numbers are even numbers that differ by the constant of 2, so “6” is the correct answer. | IQ | affiliative bond of teacher-student | β=0.314→*r*=0.36 | the affiliative bond contributed significantly to the increase in INS | fNIRS | WTC | 0.04-0.05Hz | the right sensorimotor cortex | Chinese |
| 22 | Pan  2021 | 16 | FTF | HTF | mathematical knowledge | Participants were taught numerical reasoning strategies to find the hidden rules and relations within a digit sequence. For example, for a given digit sequence “1, 3, 5, __, 9”, the hidden rule is that all digits in the sequence are odd numbers that differ by the constant of “2”; as a result, “7” is the correct answer. | post VS. pre | 20-items in pre & post test | 0.62 | correlations of INS in cluster1 and the learner's improvement was significant. | fNIRS | WTC | 0.16-0.19Hz | left inferior frontal cortex | Chinese |
|  | Pan  2021 | 16 |  |  |  |  |  |  | 0.38 | correlations of INS in cluster3 and the learner's improvement in numerical was significant. | fNIRS | WTC | 0.16-0.19Hz | left inferior frontal cortex | Chinese |
| 20 | Meshulam  2021 | 24 | / | / | conceptual knowledge | the course introduces basic concepts in programming and computer science | post VS. pre | Final exam: self-paced, with exam questions (16 in total) spanning a variety of course topics from programming to theory. By the end of the course, all students demonstrated knowledge gains (pre-post comparison). | 0.62 | prediction of exam scores from INS in different ROIs | fMRI | ISC | / | angular gyrus | English |
|  | Meshulam  2021 | 24 |  |  |  |  |  |  | 0.53 |  |  |  |  | anterior cingulate |  |
|  | Meshulam  2021 | 24 |  |  |  |  |  |  | 0.75 |  |  |  |  | hippocampus |  |
|  | Meshulam  2021 | 24 |  |  |  |  |  |  | 0.4 |  |  |  |  | post. sup. temporal gyrus |  |
|  | Meshulam  2021 | 24 |  |  |  |  |  |  | 0.61 |  |  |  |  | precuneus |  |
|  | Meshulam  2021 | 24 |  |  |  |  |  |  | 0.29 |  |  |  |  | amygdala |  |
|  | Meshulam  2021 | 24 |  |  |  |  |  |  | 0.46 |  |  |  |  | early auditory |  |
|  | Meshulam  2021 | 24 |  |  |  |  |  |  | 0.41 |  |  |  |  | early visual |  |
| 23 | Zhu  2021 | 24 | FTF | HTF | conceptual knowledge | A set of ten psychology concepts from the topic of judgement and decision making. Each concept has a term, a one-sentence definition and two examples. | post VS. pre | Pre-testing: learners were required to match 10 definitions with 10 terms from provided 12 terms. Post-testing:　learners had to match 10 novel examples with 10 terms from provided 12 terms. Relative accuracy was computed by subtracting z-score of the accuracy on pre-testing from that on post-testing. | 0.57 | INS on Cluster 3 could successfully predict learners’ relative accuracy | fNIRS | WTC | 0.018–0.027 Hz | left postcentral gyrus and superior parietal gyrus | Chinese |

N, sample size. IS, Interaction Style. IM: Interaction Mode. MT: Measuring technique. CM of INS, Calculation method of INS. FTF, face-to-face. NFTF, non-face-to-face. HTF, High turn-taking frequency. LTF, low turn-taking frequency. Post, post-testing scores. Post VS. Pre, comparison between post-testing and pre-testing scores. IQ, interaction quality evaluation scores. fNIRS, functional near-infrared spectroscopy. EEG, electroencephalography. fMRI, functional magnetic resonance imaging. TI, total interdependence. WTC, Wavelet transform coherence. ISC, inter-subject correlation. “/” indicates the coding is not applicable for the sample. We used two colors in the table to distinguish the independent samples, and the effect sizes under the same independent sample were *combined*.
